# Globe rupture: a single-center retrospective study of demographic patterns and outcomes

**DOI:** 10.1038/s41598-020-76121-2

**Published:** 2020-11-05

**Authors:** Janejit Choovuthayakorn, Susama Chokesuwattanaskul, Phit Upaphong, Pongsant Supreeyathitikul

**Affiliations:** Department of Ophthalmology, Faculty of Medicine, Chiang Mai University, 110 Intavaroros Road, Maung, Chiang Mai 50200 Thailand

**Keywords:** Medical research, Vision disorders

## Abstract

Globe rupture is one of the severe mechanisms of eye injury. This study aimed to describe an epidemiologic patterns and visual outcomes of the open globe injury from globe rupture at a tertiary referral centre. Medical records of 167 patients (173 eyes) were retrospectively reviewed. Overall, males were predominant (82%). Road traffic- (26.3%) and work- (23.4%) were the major contributors. However, falling was the main mechanism in the elderly aged over 60 years. At presentation, 91.4% of eyes had initial visual acuity (VA) of worse than 20/200. The mean (SD) VA in logarithm of the minimum angle of resolution (LogMAR) unit at final [1.8 (1.1)] was significantly improved from presenting VA [2.4 (0.6)] (p ˂ 0.001). Predictors for final VA of worse than 20/400 included poor initial VA, presence of relative afferent pupillary defect, and endophthalmitis. In conclusion, this study observed the peak incidence of globe rupture in young teen to early adult patients, with unique characteristics in each age group. Even with significant visual improvement following the treatments, profound visual loss was still a common consequence. Thus, the role of effective prevention along with a multidisciplinary team together with timely and prompt ophthalmic management should be emphasised.

## Introduction

A mechanical ocular trauma, in particular open globe injury (OGI), is one of the global ophthalmic conditions leading to hospitalisation and subsequent severe visual impairments^1–7^. Considering the mechanisms of open globe injury, different objects will damage the globe in different manners. As classified by Birmingham Eye Trauma Terminology System (BETT), OGI caused by a blunt object (or an impaction with an inside-out mechanism) is defined as globe rupture, while, OGI caused by a sharp object (or a laceration with an outside-in mechanism) is defined as either penetration, perforation, or intraocular foreign body (IOFB)^8^. The incidence of OGI varies from study to study, which may suggest the unique characteristics of each population and also activities at risk^9–13^. In previous OGI reports, patients who sustained globe ruptures had less favourable visual outcomes in comparison with those of lacerations^11–15^. However, the research focusing on clinical characteristics and visual outcomes of patients following the rupture is insufficient to implement specific clinical guidelines and safety measurements. Our study, therefore, aims to explore an epidemiologic pattern, including demographics and activities at risk, and visual outcomes of the patients with globe rupture. This information is essential to effectively guide the medical counselling for patients with globe rupture.

## Material and methods

This retrospective study was conducted in accordance with the tenets of the Declaration of Helsinki and was approved by the Research and Ethics Committee, Faculty of Medicine, Chiang Mai University. With the anonymous data extraction, an informed consent was waived. The clinical database of all patients, admitted for OGI between Jan 1, 2006 and Dec 31, 2016, were reviewed. Only the OGI patients with identified mechanisms of injury as globe rupture (e.g. a full-thickness eye wall disruption from a blunt trauma as defined by BETT), who had a follow-up period of at least 3 months, were further included in the study. Using a clinical record form, the patient demographics, detailed activities related to the injury, ophthalmic examinations and treatments, were reviewed. The ophthalmic examinations included presenting and final visual acuity (VA) from the most recent visit available; anterior segment findings by slit lamp examination; posterior segment abnormalities whether assessed by indirect ophthalmoscopy, B-scan ultrasonography, or other imaging modalities; and adnexal injury. The Ocular Trauma Score (OTS) was also determined from the clinical records.

### Statistical analysis

The patient demographic data was descriptively presented as either mean and standard deviation (SD), median and interquartile range (IQR), or frequency (as percentage). Snellen VA was converted to LogMAR (logarithm of the minimum angle of resolution) unit for statistical analysis. The conversion of counting finger (FC), hand motion (HM), light perception (PL), no light perception (NPL) to logMAR was based on Reed G’s report^16^. An unfavourable visual outcome was defined as a final VA of worse than 20/400. A multivariable regression analysis for unfavourable final visual outcome was analysed using the Backward LR regression method, adjusted for age, gender, initial VA, zone of injury, presence of uveal tissue protrusion, presence of vitreous prolapse, presence of retinal and choroidal detachment, presence of relative afferent pupillary defect (RAPD), presence of endophthalmitis, and time interval from injury to the hospital. An eye with anatomic restoration is defined as an eye with flattened retina within the major vascular arcade, and vice versa, an eye with anatomic non-restoration is defined as an eye either with retinal detachment within the major vascular arcade area, or being phthisis or enucleated before the final clinical visit. A *p* value of ˂ 0.05 was considered significant.

## Results

From a total of 978 patients (998 eyes) who sustained OGI over the study period, globe rupture occurred in 167 patients (173 eyes, 17.3%) with a mean (SD) follow-up time of 17.8 (24.4) months. Among these ruptured cases, 8 patients acquired bilateral eye injuries (6 patients had globe ruptures in both eyes, and 2 patients had globe rupture in one eye and globe penetration in the other). Bilateral eye injuries occurred following road traffic-related accidents in 4 cases, firework-related accidents in 2 cases, and weapon-related explosions in 2 cases. Overall, males were predominant (137/167, 82.0%) with a mean (SD) age of 36.8 (17.2) years, ranged from 2 to 90 years, whereas 30/167 (18.0%) patients were female with a significant higher mean (SD) age of 45.1 (24.8) years, ranged from 1 to 82 years (*p* = 0.03).

**Figure 1:**
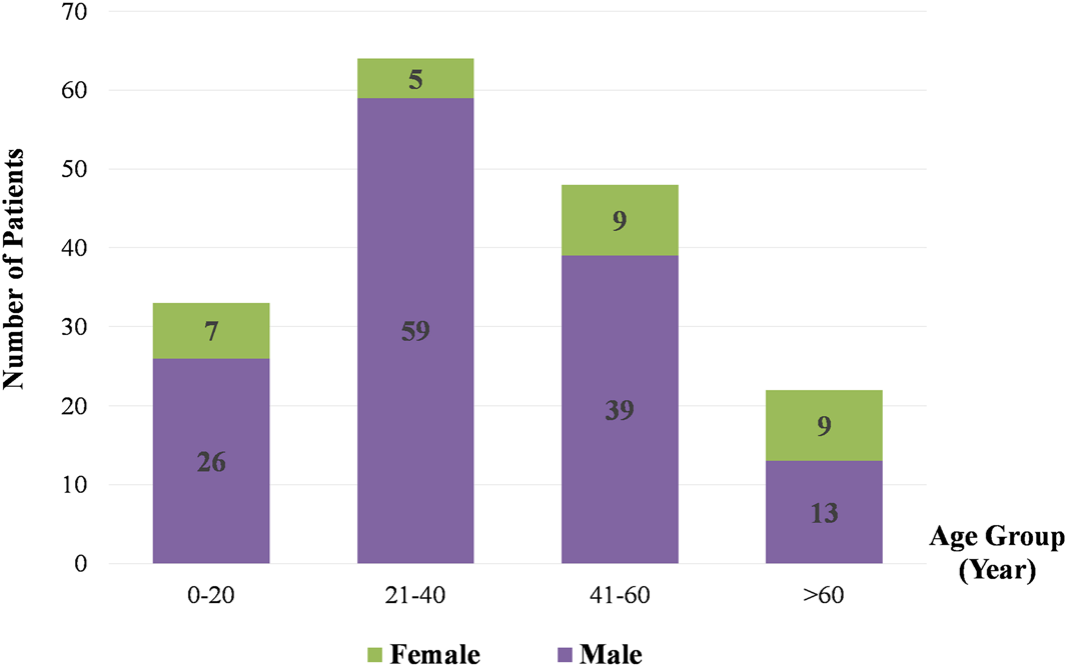
The incidence of globe rupture by genders and age groups.

A peak incidence of globe rupture was found in patients aged between 21 and 40 years. The highest proportion of female to male patients was observed in the elderly group (Fig. 1). Most common circumstances leading to globe rupture were road traffic-related accidents (44/167, 26.3%), followed by work- (39/167, 23.4%), and weapon-related injuries 18/167 (10.8%) (see Table 1). In patients aged over 60 years, falling was a major event leading to ruptured globe.

**Table 1:** Distribution of activities causing globe rupture divided by age groups.

Activities/settings	Number (%)	Age (year), number			
		≤ 20	21—40	41—60	˃ 60
Road traffic-based	44 (26.3)	10	21	9	4
Work-based	39 (23.4)	1	18	14	6
Weapon-related accident	18 (10.8)	3	11	4	0
Assault	18 (10.8)	5	6	6	1
Firework	16 (9.5)	6	4	6	0
Recreation	12 (5.4)	8	0	3	1
Fall	9 (5.4)	0	0	2	7
Home-based	6 (3.6)	0	1	2	3
Sports	5 (3.0)	0	3	2	0
Total	167	33	64	48	22

The median time (IQR) from injury to the hospital was 24 (6–120) h. Among these, 46/173 (26.6%) eyes were presented within the first 6 h, and 44/173 (25.4%) eyes arrived at the hospital between 6 and 24 h after injury. The majority of injured eyes 163/173 (91.4%) had initial VA of worse than 20/200. Of those with sufficient data available for OTS classification, 159/173 (91.0%) eyes were classified as severe injury with OTS class I and class II, while 8/173 (4.6%) eyes as OTS class III. None were classified into OTS class IV and V. Ophthalmic characteristics of globe rupture are demonstrated in Table 2. Notably, the eye wall dehiscence at previous surgical sites occurred in 9/173 (5.2%) eyes. Endophthalmitis was present in 8/173 (4.6%) eyes.

**Table 2:** Presenting ophthalmic characteristic of ruptured eyes.

Characteristics	Eyes (%)
**Zone of injury**	
I	38 (22.0)
II	71 (41.0)
III	64 (37.0)
**Wound length**	
** ≤ **10 mm	104 (60.1)
** > **10 mm	66 (38.2)
Unknown	3 (1.7)
Presence of RAPD	82 (47.4)
Hyphema	113 (64.6)
**Lens injury**	
Traumatic cataract	42 (24.3)
Penetrating lens injury	16 (9.2)
Lens subluxation/dislocation	20 (11.6)
Lens extrusion	14 (8.1)
Uveal tissue prolapse	100 (57.8)
Vitreous prolapse	60 (34.7)
Vitreous hemorrhage	93 (53.8)
**Retinal injury**	
Retinal detachment	59 (34.1)
Retinal dialysis	2 (1.2)
**Adnexal injury**	
Eye lid injury	32 (18.3)
Orbital injury	11 (6.3)
Both lid and orbital injury	15 (8.6)
**Ocular trauma score**	
Class I (0–44)	91 (52.6)
Class II (45–65)	68 (39.3)
Class III (66–80)	8 (4.6)
Class IV (81–91)	0 (0)
Class V (92–100)	0 (0)
Cannot be assessed	6 (3.5)

RAPD = relative afferent pupillary defect.

For surgical management, a primary repair surgery was initially performed in every eye. However, in 9/173 (5.2%) eyes, the primary repair surgery was attempted and shifted to a primary enucleation due to unrepairable damage, whereas, in 15/173 (8.7%) eyes, the enucleation was performed in subsequent operations. Anterior segment reconstruction surgery was performed in 26/173 (15.0%) eyes, while posterior segment surgery in 104/173 (60.1%) eyes. In addition, 71/173 (41.0%) eyes underwent lens surgery.

### Anatomic and visual outcomes

**Figure 2:**
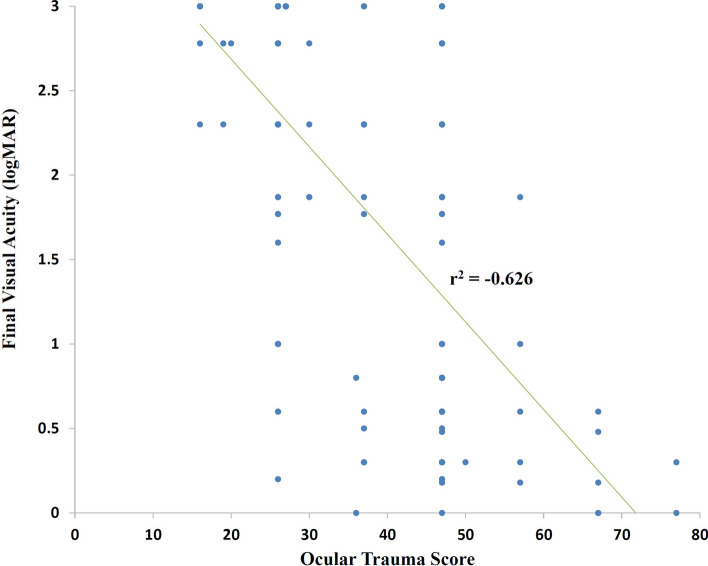
The correlation between presenting Ocular Trauma Score and final visual acuity of eyes with ruptured open globe injury.

**Figure 3:**
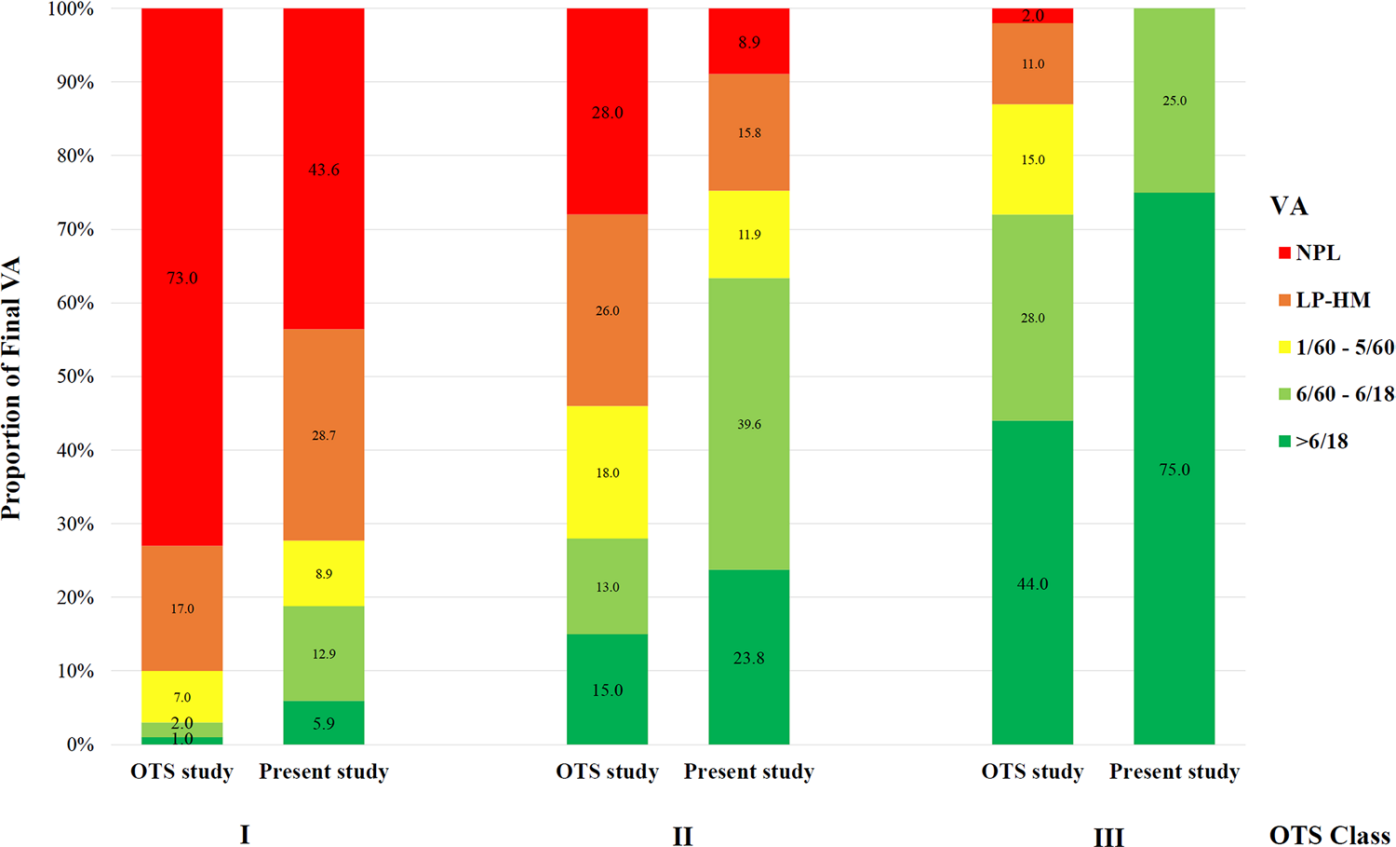
Proportion of final visual acuity of eyes categorised in each Ocular Trauma Score (OTS) classes in present study comparing to the OTS study.

Anatomic restoration was achieved in 116/173 (67.1%) eyes, with either fluid-filled [96/173 (55.5%)] or silicone oil-filled [20 (11.6%)] eyes. In anatomic non-restoration eyes with no enucleation, 28/173 (16.2%) eyes had unrepairable retinal detachments, and 3/173 (1.7%) eyes became phthisis. Regarding visual outcomes, mean final LogMAR VA was significantly improved from the presenting VA [2.4 (0.6) to 1.8 (1.1), *p* ˂ 0.001]. The final VA of 20/200 or better was achieved in 67/173 (38.8%) eyes, while 46/173 (26.6%) eyes had NPL at the final assessment (Table 3). There was a moderate negative correlation between presenting OTS and final VA (r^2^ = − 0.626, Fig. 2). Figure 3 illustrates the proportion of final VA of eyes categorised by OTS classification in comparison to the data from the OTS study^17^. For a multivariable regression analysis for final VA of worse than 20/400, poor initial VA [*p* < 0.001, odds ratio (OR) 12.672; 95% confidence interval (CI) 4.142–38.769], presence of RAPD (*p* < 0.001, OR 5.287; 95% CI 2.287–12.225), and endophthalmitis (*p* = 0.040, OR 26.516; 95% CI 4.187–113.240) were the poor predicting factors explaining 48% of the variance (R^2^ = 0.48). Secondary glaucoma was diagnosed in 12/173 (6.9%) eyes.

**Table 3:** Distribution of presenting and final visual acuity of eyes with ruptured globe injury.

	Initial visual acuity, number (%)	Final visual acuity, number (%)
20/40 and better	2 (1.1)	29 (16.8)
20/50 to 20/200	7 (4.0)	38 (22.0)
19/200 to projection of light	129 (74.6)	57 (32.9)
No perception of light	29 (16.8)	46 (26.6)
Cannot be assessed	6 (3.5)	3 (1.7)

## Discussion

This study revealed that globe rupture commonly occurred in young males, aged between 21 and 40 years. Most of the incidences were related to road traffic-related accidents and injuries at the workplace. Despite significant visual improvements observed following treatment, approximately one-fourth of the patients (26.6%) remained NPL vision at the final follow-up visit, thus, implying the high severity in nature of globe rupture with extensive ocular tissue damage. Consistent with other studies, poor final VA was associated with poor initial VA and presence of either RAPD or endophthalmitis.

In literature, the reported incidence of globe rupture among OGI studies varies from 13 to 69%^5–7,11–14^. This phenomenon is partly explained by the different age groups of patients included in the studies, in which the data with higher mean age of OGI patients tended to present higher percentages of globe rupture^6,7,12^. A study from Japan by Fujikawa et al*.* reported a mean age of OGI patients of 51 years in which globe rupture represented about two-thirds (69%) of the cases^12^. On the contrary, a study from New Zealand by Court et al. reported a lower median age of patients with OGI of 37 years, consequently, the globe rupture was reduced to approximately one-third (36%) of the cases^7^. Also, a study from Turkey by Ustaoglu et al*.* reported only 13% of the cases were globe rupture with the mean age of overall OGI patients at 33 years old^6^. Consistent with our study, as the median age of OGI patients was 39 years old, globe rupture contributed to only 17% of the overall cases. Moreover, the inhomogeneous distribution of affected age groups may reflect different daily activities of the at risk populations, therefore the demographic data should be taken into consideration when implementing the safety measurements in each country.

Some specific circumstances were more likely to cause globe rupture than others^18–23^. A study by Feng et al. reported that violence, explosion, and traffic were the most frequent causes leading to severe ruptured eyes requiring vitreoretinal surgery^24^. In accordance with a series by Burstein et al., violence was the main causative mechanism^25^. In this study, road traffic, work, and falling were the major circumstances at risk, however, distinct patterns can be observed in different age groups. Additionally, a study of Morikawa et al. demonstrated a significantly higher proportion of globe rupture (91%) in fall-related OGI patients, predominantly in the elderly women group, compared to the non-fall-related group (38%)^23^. Our study also confirmed that falling was the situation with a high susceptibility of globe rupture, particularly in the elderly^23,26–28^. Moreover, approximately 5% of ruptured eyes in this study occurred at a previous surgical site. Under any circumstances, patients with previous intraocular surgeries should, therefore, be reminded of their additional life-long risk of globe rupture^27,28^.

For visual outcomes, in a study by Feng et al., nearly 40% of ruptured eyes treated with vitreoretinal surgery achieved anatomic restoration, still 19% had NPL vision. An extensive retinal loss, closed-tunnel retinal detachment, proliferative vitreoretinopathy grade C, and choroidal damage were the predictors for unfavourable visual outcomes^24^. In eyes with scleral rupture limited to zone II and zone III, Yucel et al. observed that as high as 42.6% of eyes resulted in final vision of NPL. Poor final VA was significantly associated with poor presenting VA, horizontal midline wound, longer wound length, presence of hyphema, vitreous hemorrhage or retinal detachment, and no cataract surgery^29^. In this study, approximately one-fourth (26%) of eyes had NPL. In comparison to the original report from the OTS study in 2002, more eyes in the poor initial OTS class had better VA gain following the treatments^17^. This may partly be explained by the improvement of ophthalmic surgeries and treatments over the decade and may warrant the review of OTS classification in predicting the final visual outcomes. The poor prognostic characteristics were poor presenting VA, presence of RAPD, and endophthalmitis. Another significant characteristic of OGI patients in this study was time to presentation, as half of the cases presented at the hospital after 24 h from the time of injury.

The major limitation of this study was in its retrospective nature which compromised an extraction of some details of the injuries and the clinical characteristics.

In conclusion, this study shows epidemiological data of globe rupture. Better visual outcomes in these severely damaged eye injuries have been achieved compared to the eyes in the original OTS study. This study identified traffic accidents, work, and falling as the circumstances at risk of globe rupture. In terms of clinical assessment and medical counselling, the poor prognostic characteristics leading to unfavourable visual outcomes, including presenting VA, presence of RAPD, and endophthalmitis, should be primarily identified and prompt intensive management.
